# Association of an In-House Blood Bank with Therapy and Outcome in Severely Injured Patients: An Analysis of 18,573 Patients from the TraumaRegister DGU®

**DOI:** 10.1371/journal.pone.0148736

**Published:** 2016-11-03

**Authors:** Florian Debus, Rolf Lefering, Philipp Lechler, Tim Schwarting, Benjamin Bockmann, Erwin Strasser, Carsten Mand, Steffen Ruchholtz, Michael Frink, TraumaRegister DGU

**Affiliations:** 1 Department of Trauma, Hand and Reconstructive Surgery, University Hospital Gießen and Marburg, Marburg, Germany; 2 Institute for Research in Operative Medicine (IFOM), University of Witten/Herdecke, Cologne, Germany; 3 Committee on Emergency Medicine, Intensive Care and Trauma Management of the German Trauma Society (Sektion NIS), Berlin, Germany; 4 Department of Transfusion Medicine and Hemostaseology, University Hospital Erlangen, Erlangen, Germany; Georgia Regents University Cancer Center, UNITED STATES

## Abstract

**Introduction:**

Hemorrhagic shock remains one of the most common causes of death in severely injured patients. It is unknown to what extent the presence of a blood bank in a trauma center influences therapy and outcome in such patients.

**Material and Methods:**

We retrospectively analyzed prospectively recorded data from the TraumaRegister DGU® and the TraumaNetzwerk DGU®. Inclusion criteria were Injury Severity Score (ISS) ≥ 16, primarily treated patients, and hospital admission 2 years before or after the audit process.

**Results:**

Complete data sets of 18,573 patients were analyzed. Of 457 hospitals included, 33.3% had an in-house blood bank. In trauma centers with a blood bank (HospBB), packed red blood cells (PRBCs) (21.0% vs. 17.4%, p < 0.001) and fresh frozen plasma (FFP) (13.9% vs. 10.2%, p <0.001) were transfused significantly more often than in hospitals without a blood bank (Hosp0). However, no significant difference was found for in-hospital mortality (standard mortality ratio [SMR, 0.907 vs. 0.945; p = 0.25). In patients with clinically apparent shock on admission, no difference of performed transfusions were present between HospBB and Hosp0 (PRBCs, 51.4% vs. 50.4%, p = 0.67; FFP, 32.7% vs. 32.7%, p = 0.99), and no difference in in-hospital mortality was observed (SMR, 0.907 vs. 1.004; p = 0.21).

**Discussion:**

In HospBB transfusions were performed more frequently in severely injured patients without positively affecting the 24h mortality or in-house mortality. Easy access may explain a more liberal transfusion concept.

## Introduction

Despite continual improvement in the treatment of severely injured patients, major injuries remain the most common cause of death in patients less than 45 years of age [1]. Because severely injured patients are commonly young and healthy, the medical and socio-economic consequences can be devastating. Hemorrhagic shock is an independent factor for outcome and survival of severely injured patients [2]. Hemorrhagic shock has been reported to cause up to 50% of all fatalities after major injuries [3, 4], especially during the early period after trauma [5].

Obvious hemorrhagic shock frequently requires massive transfusion, which has been shown to be associated with an adverse outcome [6]. Therefore, protocols for massive transfusion have been developed and implemented in treatment algorithms [7–9]. Therapeutic concepts in severely injured patients with hemorrhagic shock have been noted to include administration of coagulation-supporting drugs without reducing the number of transfused packed red blood cells (PRBCs) [10, 11]. Although the infrastructure of trauma centers has continued to improve during the last decades, no evidence-based recommendations regarding transfusion management facilities, such as blood banks or blood depots, are available.

The present study investigates the influence of an in-house blood bank on transfusions and outcome in severely injured patients.

## Material and Methods

The TraumaRegister DGU® (TR-DGU), of the *Deutsche Gesellschaft für Unfallchirurgie*, was founded in 1993 with the aim of achieving anonymous, standardized documentation of patients suffering from severe injuries. Data are prospectively collected in four consecutive time phases from the time of the accident until discharge from hospital. The documentation includes detailed information on demographics; injury pattern; comorbidities; pre- and in-hospital management; intensive care unit (ICU) course; and relevant laboratory findings, including transfusion and outcome data for each individual. The inclusion criteria are hospital admission via emergency room with subsequent ICU care; or arrival at the hospital with vital signs but death before admission to the ICU.

Data are anonymously submitted, by qualified personnel using a web-based software at participating hospitals, to a central database. The present study was conducted in accordance with the publication guidelines of the TR-DGU and registered as TR-DGU project identification (ID) 2013–055.

For this analysis, data from the TR-DGU and the TraumaNetzwerk DGU® (TN-DGU) were combined. During the certification process, various structural and institutional parameters were recorded during hospital audits and documented in a central data bank. These hospitals were divided in two subgroups: Hospitals with blood bank (HospBB) and without blood bank (Hosp0). The data banks were connected, so all cases available from the TR-DGU were examined considering these two subgroups. Inclusion criteria were Injury Severity Score (ISS) ≥ 16, treatment in a German trauma center, and primary admission from the scene. Furthermore, only patients treated 2 years before or after the date of audit were included.

Various parameters were compared, including demographic data, mechanism of injury, injury pattern and severity, status on admission, transfusions performed, treatment in an ICU, and outcome. Data are documented in the TR-DGU using two different forms. The standard documentation form has 100 items and the quality management form has 40 items. The parameters prothrombin time (PT), sepsis, and multi-organ failure (MOF) were available only on the standard documentation form. For a more transparent presentation, the valid [n] for every item on which statistical tests are based is provided. With regard to transfusions performed, only blood products administered during the initial resuscitation period until admission on ICU were considered. Additional analyses were performed for patients with clinically apparent shock at the time of admission.

Within the TNW-DGU, hospitals were categorized as a local (LTC), regional (RTC), or supraregional trauma center (STC), depending on their level of care. This classification system is based on defined structural and organizational requirements, which were surveyed during the certification process.

Observed mortality rates were compared with the Revised Injury Severity Classification-II (RISC-II) prognosis, and standardized mortality ratio (SMR) was calculated as the ratio of observed and expected mortality years. The RISC-II was developed to calculate the probability of death in patients suffering major trauma and included variables such as worst and second-worst injury, age, sex, mechanism of injury, and laboratory and physiological parameters [12, 13]. We analyzed in-hospital mortality as well as 24-h mortality as markers for death due to bleeding and hemorrhagic shock.

To ensure exclusion of hospital effects on in-hospital mortality, a multivariate logistic regression was performed. The independent variables of the logistic regression analysis were RISC II score (to adjust for patient characteristics), hospitals (to adjust for hospital effects), presence of shock on admission, and availability of a blood bank. Results are reported as odds ratio (OR) with 95% confidence interval (CI) and p value.

The present study follows the publication guidelines of the TR-DGU. Anonymity of hospital data and individual patient data were guaranteed. According to the guidelines of the local ethics committee, for retrospective studies, no formal approval was required.

Analyses were performed using IBM SPSS Statistics for Windows, Version 22.0 (IBM Corp., Armonk, NY, USA). A p value < 0.05 was considered statistically significant. However, because the large number of cases led to significant p values even in cases of minor differences without clinical relevance, significant differences should be interpreted with caution. The Mann-Whitney U test and Chi-squared test were used to compare continuous and categorical variables, respectively.

## Results

### Basic data

In the current analysis, structural data for 457 hospitals (250 [54.7%] LTCs, 173 [37.9%] RTCs, and 69 [15.1%] STCs were available. Overall, 152 hospitals (33.3%) housed a blood bank. Inclusion criteria were met by 18,573 patients. Because of the small number of patients treated in LTCs (without blood bank, n = 174; with blood bank, n = 782), further analyses were limited to RTCs and STCs. In RTCs, 2,463 of 5,522 patients (44.6%) were treated in HospBB; in STCs, 10,525 of 12,095 patients (87.0%) were treated in HospBB.

Hosp0 stored a limited number of units of 0-Rh-negative PRBCs (mean, 6.6 units [LTCs, 4.6; RTCs, 7.9; and STCs, 10.5 units]).

Multivariate logistic regression analysis showed that shock on admission tended to have a negative effect on in-hospital mortality (OR, 1.13; CI, 0.98–1.30; p = 0.11) and that the presence of a blood bank tended to have a positive effect on in-hospital mortality (OR, 0.47; CI, 0.002–9.05; p = 0.62). However, neither result reached statistical significance. The hospital ID had no effect on mortality.

### Analysis of all documented patients

Patient age, ISS, and physiological and laboratory parameters showed only minor differences between groups (Table 1).

**Table 1:** Age, ISS, and Physiological and Laboratory Parameters on Admission for All Patients.

	Valid (n)	Hosp0 n = 305	HospBB n = 152	p-value
**Patients (n)**	17.617	4.629	12.988	< 0.001
**treated in STCs**		1.570	10.525	
**treated in RTCs**		3.059	2.463	
**Age (years)**	17.505	49.6 / 49.0 (30–60)	47.2 / 47.0 (28–65)	< 0.001
**ISS**	17.617	27.0 / 25.0 (18–33)	28.4 / 25.0 (19–34)	< 0.001
**sBP on admission (mmHg)**	15.925	123 / 120 (105–140)	121 / 120 (100–140)	< 0.001
**Hb on admission (g/dl)**	16.374	12.5 / 12.9 (11–14)	12.0 / 12.4 (10–14)	< 0.001
**Prothrombin time on admission (%)**	15.682	81.7 / 87.0 (71–98)	78.9 / 83.0 (66–96)	< 0.001
PTT on admission (sec.)*	9.525	34.2 / 29.2 (26–34)	35.3 / 29.7 (26–35)	< 0.001
Sepsis (%)*	9.590	8.3	8.9	0.431
MOF (%)*	9.624	29.6	34.6	< 0.001

* Available only on standard documentation form

Data presented as “mean / median (interquartile range)”. Hb, hemoglobin; ISS, injury severity score; MOF, multi-organ failure; PTT, partial thromboplastin time; sBP, systolic blood pressure.).

Blunt trauma represented the predominant mechanism of injury (n = 16,108 [96.1%[) with no significant between-group difference observed (Hosp0: n = 4,110 [96.3%] vs. HospBB: n = 11,998 [96.1%]; p = 0.51). Motor vehicle accident was the mechanism of injury in 9,684 cases (59.0%), with no significant difference between Hosp0 (n = 2,487 [58.7%]) and HospBB (n = 7,197 [59.1%]) p = 0.78). Relevant abdominal injuries (Abbreviated Injury Scale (AIS) ≥ 3) were found in 3,039 cases (17.3%), with significantly more observed in Hosp0 (n = 827 [17.9%]) than in HospBB (n = 2,212 [17.0%]) p = 0.022). Relevant extremity injuries (AIS ≥ 3) represented 6,056 (34.4%), and significantly more were observed in Hosp0 (n = 1,640 [35.4%]) than in HospBB (n = 4,416 [34.0%]), p = 0.017). Severe head injuries (AIS ≥ 3) were sustained by 9,482 patients (53.8%). A significantly greater number of these patients were treated in HospBB (n = 7,198 [55.4%]) than in Hosp0 (n = 2,284 [49.3%]) p<0.001). Accordingly, patients with an initial Glasgow Coma Scale (GCS) score of ≤ 8 (n = 5,341 [32.0%]) were more frequently treated in HospBB (n = 4,189 [34.0%]) than without (n = 1,152 [26.3%]) p<0.001). Severe injuries of the chest (AIS ≥3) were found in 10,218 patients (58.0%). These patients were treated significantly more frequently in hospitals Hosp0 (n = 2,763 [59.7%]) than in HospBB (n = 7,455 [57.4%]) p<0.001).

#### Transfusion of blood products

In 3,495 patients (20.0%), one or more units of PRBCs were transfused during the initial resuscitation period. Transfusions of PRBCs were performed more frequently in in HospBB than in Hosp0 (Table 2).

**Table 2:** Frequency of Administration of Blood Products in All Patients and in Patients with Shock on Admission.

	Hosp0%(n)	HospBB %(n)	
**All Patients**			
***Administration of PRBC***	17.4% (800)	21.0% (2695)	p<0.001
***Administration of >10 units PRBC***	3.2% (147)	5.1% (650)	p<0.001
***Administration of FFPs***	10.2% (469)	13.9% (1793)	p<0.001
**Patients with shock on admission**			
***Administration of PRBC***	51.4% (291)	50.4% (889)	p = 0.67
***Administration of >10 units PRBC***	14.0% (79)	18.4% (325)	p = 0.015
***Administration of FFPs***	32.7% (185)	32.7% (577)	p = 0.99

FFP, fresh frozen plasma; PRBCs, packed red blood cells.

**Fig 1:**
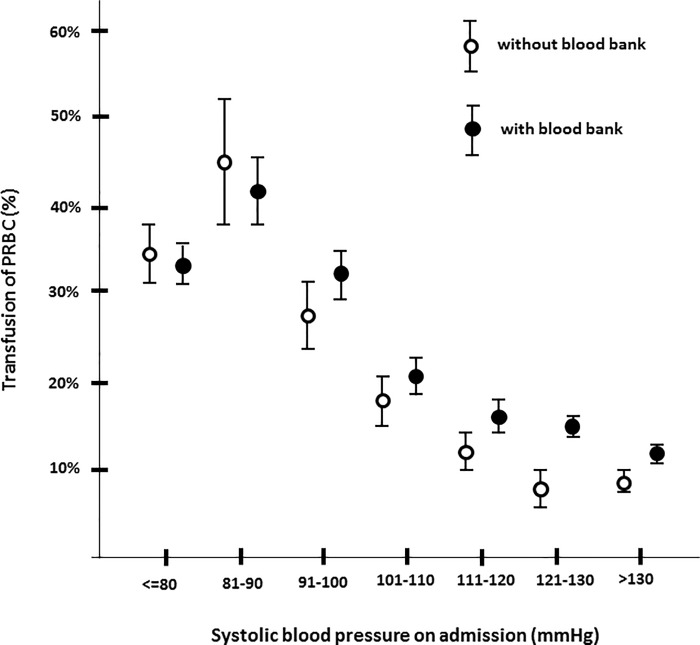
Frequency of transfusion of PRBCs (%) in relation to the systolic blood pressure (mmHg) on admission. Data is presented as mean with 95% confidence intervals, PRBC, packed red blood cell.

The rate of transfusion was significantly greater in HospBB, particularly for patients without shock on admission (blood pressure >90 mmHg) (Fig 1). A massive transfusion, defined as donation of ≥10 units PRBCs during the initial resuscitation period until admission on ICU, was more frequently performed in HospBB. Fresh frozen plasma (FFP) was transfused less often in Hosp0 (Table 2). No differences were found in additional supportive coagulation management (e.g., administration of fibrinogen and the prothrombin complex PPSB (prothrombin, proconvertine, Stuart factor, and anti-hemophilia B factor)) between groups (data not shown).

Mean number of units of PRBCs transfused per patient was 1.1±3.4 (median value 0.0) in Hosp0 and 1.6±5.0 (median value 0.0) in HospBB (p<0.001). Accordingly, significantly less FFP was administered in Hosp0 (0.7±3.1 units, median value 0.0) than in HospBB (1.1±4.2 units, median value 0.0) (p<0.001).

#### Influence on outcome

In the present analysis, all 17,457 patients for whom a RISC-II-Score could be evaluated were included. Patients treated in Hosp0 had a mortality of 17.7% (807 of 4,568) while mortality was 19.7% (2,538 of 12,889) in patients treated in HospBB. Mortality during the first 24 h was comparable between groups (Hosp0 9.5%, vs. HospBB 11.0%; p = 0.05). With regard to expected mortality, as calculated by the RISC-II score, increased survival was observed in both groups. The SMR was comparable between both groups (Table 3).

**Table 3:** Comparison of Observed and Expected Mortality in All Patients.

	Hosp0	HospBB
**Observed mortality**	17.7%	19.7%
**CI 95%**	16.6–18.8	19.0–20.4
**Expected mortality based on RISC II**	18.7%	21.7%
**Standardized mortality rate**	0.945	0.907
**CI 95%**	0.886–1.004	0.876–0.939
**p-value**	0.2515	

CI, confidence interval.

Patients treated in HospBB suffered MOF significantly more often, than those treated in Hosp0; no significant difference was observed in the incidence of sepsis (Table 1).

### Analysis of patients suffering from shock on admission

In an additional analysis, patients with shock, defined as a systolic blood pressure ≤ 90 mmHg, on admission were evaluated. Patients with shock (n = 2,424) showed only minimal differences in incidences of MOF and sepsis (Table 4).

**Table 4:** Age, ISS, and Physiological and Laboratory Parameters on Admission in Patients with Clinically Apparent Shock.

	Valid (n)	Hosp0	HospBB n = 152	p-value
		n = 305		
**Patients (n)**	2424	585	1839	≤ 0.001
**Age (years)**	2401	50.5 / 52.0 (31–70)	46.9 / 46.0 (28–65)	≤ 0.001
**ISS**	2424	35.7 / 34.0 (24–45)	37.2 / 34.0 (25–48)	0.075
**sBP. on admission (mmHg)**	2424	70 / 80 (60–90)	69 / 80 (60–90)	0.37
**Hb on admission (g/dl)**	2209	10.3 / 10.6 (8.2–12.5)	9.8 / 9.8 (7.6–12.1)	≤ 0.001
**Prothrombin time on admission (%)**	2067	64.9 / 68.0 (47–85)	62.7 65.0 (43–82)	0.056
PTT on admission (sec.)*	1134	49.4 / 33.0 (28–45)	49.7 / 35.0 (29–52)	0.059
Sepsis (%)*	1254	14.3	16.9	0.336
MOF (%)*	1127	55.3	60.6	0.133

* Available only on standard documentation form

Data presented as “mean / median (interquartile range). Hb, hemoglobin; ISS, injury severity score; MOF, multi-organ failure; PTT, partial thromboplastin time; sBP, systolic blood pressure.

#### Transfusion of blood products in patients with shock on admission

One or more units of PRBCs were transfused in 1,180 patients (50.6%) with clinically apparent shock. In this subgroup, transfusions of PRBCs were comparable between HospBB and Hosp0.

(Fig 1). The frequency of massive transfusion was significantly greater in HospBB than in Hosp0. There was no significant difference in the frequency of FFP transfusion (Table 2) or additional coagulation management (data not shown).

There was no significant difference in units of PRBCs transfused per patient between Hosp0 (3.9±6.4 units) and HospBB (5.0±9.1 units) (p = 0.34), and no significant difference in the number of units of FFP transfused between Hosp0 (2.5±5.2 units) and HospBB (3.3±7.1 units) (p = 0.49).

#### Influence on the outcome of patients with shock on admission

Mortality of patients with clinically apparent shock was 44.2% (254 of 575) in hospitals without a blood bank and 45.7% (833 of 1,821) in HospBB. There were no significant between-group differences in SMR during hospitalization (Table 5) or mortality during the first 24 h (without blood bank, 33.3%; with blood bank, 34.7%; p = 0.53).

**Table 5:** Comparison of Observed and Expected Mortality for Patients with Clinically Apparent Shock on Admission.

	Hosp0	HospBB
**Observed mortality**	44.2%	45.7%
** CI 95%**	40.1–48.2	43.5–48.0
**Expected mortality based on RISC II**	44.0%	48.6%
**Standardized mortality rate**	1.004	0.907
** CI 95%**	0.912–1.096	0.894–0.988
**p-value**	0.2123	

CI, confidence interval; RISC, Revised Injury Severity Classification-II.

In this subgroup analysis, no between-group differences in duration of mechanical ventilation required or in incidences of MOF and sepsis were observed.

## Discussion

The term blood bank, coined by Fantus in 1937 [14] to describe an area within the hospital for the storage of blood containers, currently reflects the dynamic process, performed by specially qualified staff, of producing, testing, and storing blood products. A blood bank also includes a specialized laboratory for blood-group testing to ensure ABO-compatible blood transfusion, crossmatch, alloantibody detection, and other pre-transfusion compatibility testing [15]. The blood bank must be differentiated from the blood depot, which is an autonomous organization unit that stores and provides to hospitals and outpatient clinics only blood components or plasma derivatives.

The influence of hemorrhagic shock on the outcome of severely injured patients has been shown [16, 17]. Our data are consistent with these results, demonstrating significantly increased mortality, from 19.2% to 45.3% (p<0.001), in patients with clinically apparent shock on admission. Diagnostic testing and treatment of trauma-induced coagulation disorders are complex and medically challenging. Despite stabilization of pathologic parameters [18], immediate access to a variety of blood products and other coagulation-supporting medications is required [19]. The administration of PRBCs, FFP, fibrinogen, thrombocyte concentrates, tranexamic acid, prothrombin-complex concentrates, and desmopressin represent the current standard [20–23].

In particular, infrastructural standards for transfusion of PRBCs, FFP, and other human blood products are extremely complex. Most countries have strict legal regulations governing the administration and processing of the products, as well as standards for their storage, within the framework of the Transfusion Act. Consequently, a blood bank is usually housed only in hospitals providing maximum care, and lower-level trauma hospitals frequently use other methods for adequate management of severely injured patients in emergency situations. Various solutions, such as a blood depot or cooperation with external blood banks, are usually found in clinical practice.

### Triggers of Transfusion

A hemoglobin value below 10 g/dl or a hematocrit below 30% were traditionally considered as classic transfusion triggers in hemodynamically instable patients. In the last years, more restrictive transfusion protocols are used accepting hemoglobin values of 7g/dl in hemodynamically trauma patients [24, 25]. However, especially during the initial period, the indication for transfusion cannot be based solely on laboratory parameters in trauma patients. Rapidly available physiological parameters must be considered [26, 27]. In the last years, viscoelastic haemostatic assays were introduced in the initial treatment of trauma patients [28, 29]. Furthermore, the decision for or against transfusion is influenced by additional parameters, such as trauma mechanism and experience of the trauma team leader. According to the guidelines of the American College of Surgeons, transfusion of blood products is recommended in patients in hypovolemic shock if vital parameters do not stabilize after the administration of 2000 mL of crystalloids [30]. Because of our study design, we were unable to provide information regarding the indications for transfusion performed for each individual.

### Frequency of administered Transfusions

A fundamental question is whether HospBB transfuse more frequently because of greater availability of blood products. The present study demonstrates that blood products were more frequently transfused in HospBB than in Hosp0. This applies to the administration of PRBCs and FFP. The overall 20% transfusion rate in the present study was higher than that previously reported [31]. The minor, non-significant differences in age, gender, injury severity etc. between groups in the present study do not justify the difference in transfusion rates. In particular, severely bleeding injuries of the abdomen, thorax, and extremities (AIS ≥ 3 for all) were even more frequently observed in patients treated in Hosp0 than in HospBB. Hemoglobin values, which could serve as a transfusion trigger, were significantly lower in HospBB (12.0 g/dl) than in Hosp0 (12.5 g/dl). However, both values were above the accepted transfusion threshold. Without knowing the individual indication for transfusion in each patient, it could be assumed that the simple and rapid availability of blood products in HospBB influenced the decision for transfusion of FFP and PRBCs. This is further supported by our results, which showed that more transfusions were performed in patients not suffering from shock on admission, in HospBB than in Hosp0.

In the subgroup analysis, patients with clinically apparent shock at admission were evaluated. Results showed no significant between-group differences in injury distribution and severity, laboratory findings, and vital parameters. Hemorrhage-induced hypotension has been described as a transfusion trigger and commonly results in transfusion requirement; this was confirmed by the overall transfusion rate of 50.6% in our subgroup analysis.

Patients with clinically apparent shock tended to be more frequently treated with blood products in HospBB than in Hosp0 without reaching statistical significance. Statistical significance was, however, reached for the whole population even though the absolute difference was smaller than in the subgroup analysis. Because of the different numbers of patients analyzed in total and subgroups, a comparison of differences between the total population and the subgroup should be carefully interpreted. However, the present study focused on the influence of the structural parameter blood bank on blood-product transfusion and outcome in the total population as well as in the subgroup of patients with shock on admission.

In summary, our results demonstrate that Hosp0 were able to perform adequate treatment, including required transfusion of FFP and PRBCs, of critically ill trauma patients.

### Blood depots and transportation of PRBCs

A mean of 6.6 units of PRBC (0-Rh-negative) was stored in Hosp0. We believe there is a lack of generally accepted recommendations regarding the minimum amounts of blood products to be stored. In particular, in well-structured German trauma networks, no guidelines are available [32]. With regard to transport, storage, and maintenance of the blood cold chain, strict regulations must be fulfilled to ensure the quality and safety of blood products [33–35]. It can therefore be assumed that the system of blood depots meets the requirements in clinical practice, guaranteeing that in case of an acute emergency requiring massive transfusion, other preserves can be accessed.

### Analysis of outcomes in hospitals with and without a blood bank

It must be elucidated whether patients with apparent shock on admission have increased mortality if treated in Hosp0. In the current analysis, SMR was comparable for patient with shock on admission in both groups (HospBB and Hosp0). This is extremely interesting, considering the increased frequency of transfusions performed in HospBB. More frequent transfusions associated with the presence of a blood bank did not influence the outcome in severely injured patients, and results of a study showing an association between an increased rate of blood transfusions and increased mortality [36] were not confirmed. However, in the entire investigated population as well as the analyzed subgroup suffering from shock on admission, the presence of a blood bank, associated with an increased frequency of transfusions, did not improve survival. Because of the lack of mortality reduction and considering the risks and costs of each blood transfusion [37, 38], the indication for transfusion in severely injured patients warrants critical evaluation. The easy availability of blood products should not influence clinical decision making.

Our results showed that severely injured patients with clinically apparent shock did not have increased mortality if treated in Hosp0. The increase in SMR, to 1.004 in Hosp0 from 0.907 in HospBB, was negligible and not statistically significant. Clearly, Hosp0 had an infrastructure in place to ensure the structured and rapid transport of blood products and were able to manage patients requiring massive transfusion.

Patients treated in HospBB required a longer duration of mechanical ventilation and suffered more frequently from MOF. This may be due to the greater severity of injuries treated in HospBB than those treated in Hosp0. No between-group differences were observed in patients suffering from shock on admission; not showing a difference in injury severity.

### Limitations

The present study had several limitations. Because of the number of hospitals included in this analysis, various algorithms may be applied for treatment of polytraumatized patients. Specifically, the retrospective design prevented evaluation of the indication for each transfusion. Although a hospital effect was excluded, we only analyzed one structural parameter. Effects of additional structural parameters (e.g. presence or absence of a massive transfusion protocol) on the treatment of severely injured patients cannot be excluded. Furthermore, local trauma centers were excluded from the analysis. Registry-based studies consistently suffer from a limited number of included and documented parameters. Thus, TR-DGU does not contain detailed information about coagulation management, additional tests like ROTEM® and protocols other than those mentioned in the present study. In the present study in 96.1% a blunt trauma mechanism was identified limiting the transfer of our results to other countries. Comparison of differences between the whole population and the subgroup must be carefully interpreted; because of the power of applied statistical tests, smaller absolute differences in a greater population may reach statistical significance.

## Conclusions

HospBB perform transfusions in severely injured patients more frequently without positively influencing mortality. Easy access to blood products may explain a more liberal transfusion concept. Hosp0 require an adequate infrastructure to ensure immediate access to blood products to treat severely injured patients with comparable results.
